# Sequence analysis of a viral strain isolated for the first time in the UK, clarifies the identity of a novel species of fabavirus

**DOI:** 10.1007/s00705-026-06690-6

**Published:** 2026-07-15

**Authors:** Naomi Beddoe, Sam McGreig, Adrian Fox, Ian P. Adams, Alec Forsyth

**Affiliations:** 1 Canterbury Christ Church University, North Holmes Road, Canterbury, Kent, CT1 1QU UK; 2 Fera Science Ltd., York Biotech Campus, Sand Hutton, York, YO41 1LZ UK; 3 School of Natural and Environmental Sciences, Newcastle University, Agriculture Building, King’s Road, Newcastle Upon Tyne, NE1 7RU UK

## Abstract

For the first time sequence data of a fabavirus was identified in dahlia from the UK, which was similar to virus sequence reported from dahlia in China. Initial sequences were determined by high-throughput sequencing (HTS) using the Illumina MiSeq platform, and were verified by Sanger sequencing and 5’ and 3’ rapid amplification of cDNA ends (RACE) polymerase chain reaction (PCR). The genome comprised two RNAs that were 5,851 nucleotide (nt) and 3,468 nt long, excluding the 3’-end poly-A tails, each RNA coded for a single polyprotein. The amino acid (aa) sequence identity percentages shared with recognised *Fabavirus* members over the protease-polymerase (Pro-Pol), Large Coat Protein (LCP) and combined Coat protein (CP) regions were in range with current demarcation thresholds for a distinct *Secoviridae* species. These sequence data analyses indicate that the fabavirus sequences identified in dahlia in both China and the UK represent a novel species in the family *Secoviridae*, genus *Fabavirus*. The name dahlia fabavirus (DFV) is proposed for this putative novel species tentatively named “*Fabavirus dahliae*”.

**Supplementary Information:**

The online version contains supplementary material available at 10.1007/s00705-026-06690-6.

Originating from Central America, the genus *Dahlia* consists of 42 species, grown widely around the world for commercial, horticultural and ornamental value. Dahlias are geophytes producing tubers which enable them to overwinter, an adaptation which means they are susceptible to the accumulation of viruses. Viruses can negatively affect the growth and vigour of plants, and also damage their aesthetic appeal, therefore reducing the commercial value of a crop ([19]; Gera & Zeidan, [11]). Many viruses are regulated, monitored and controlled (Defra, no date [6]) in relation to global trade to improve biosecurity [9] and to prevent the spread of diseases (World Trade Organisation, 2026 [26]). In this respect, understanding the causes and spread of viral infections and being able to test for such is important. A range of viruses have been reported to infect dahlia plants including the caulimoviruses dahlia mosaic virus (DMV) and dahlia common mosaic virus (DCMV) which are considered sequence variants of the same species *Caulimovirus tessellodahliae*, suggested to be renamed to *Caulimovirus dahlia* (Geering et al., 2022 [10], Gnanasekaranet al., 2026 [12]); the orthotospoviruses tomato spotted wilt virus (TSWV; *Orthotospovirus tomatomaculae*) [2] and impatiens necrotic spot virus (INSV; *Orthotospovirus impatiensnecromaculae*) [22], the *Cucumovirus* cucumber mosaic virus (CMV; *Cucumovirus CMV*) [3], and the *Ilarvirus* tobacco streak virus (TSV; *Ilarvirus TSV*) (Brunt, 1968 [5]; [20, 21]).

*Dahlia variabilis* plants growing in the UK exhibited virus-like symptoms including chlorosis, mottling and small lesions. Leaf samples were collected from a highly symptomatic plant and high-throughput sequencing (HTS) was undertaken to identify the potential viral origin. A MiSeq cDNA library was created and sequenced as described by Beddoe et al.,(2024) [4]. Two contigs, identified as potentially viral, were generated from the MiSeq sequence data, the first was 6012 nt (748.079x fold coverage) in length and the second was 3,471 nt (332.6989x fold coverage).

The MiSeq data were validated using reverse transcription-polymerase chain reaction (RT-PCR), cloning and Sanger sequencing. Total RNA was extracted from fresh leaves with the RNeasy Plant Mini Kit (Qiagen), then cDNA was synthesised with an RNA to cDNA EcoDry Premix (Double Primed) kit (TakaraBio) following the manufacturer’s instructions. The primers (Supplementary data 1) were designed based on the MiSeq contigs to produce overlapping regions. The resulting amplicons were ligated into pCR2.1 TA vector (Invitrogen) then sequenced by the Sanger method (DBS Genomics). The sequences were quality controlled and aligned using Sequencher (GeneCodes) and a consensus sequence was produced for RNA1 and RNA2. The 5’ and 3’ untranslated regions (UTRs) were identified and confirmed by Rapid Amplification of cDNA Ends (RACE) PCR (5’ and 3’ SMARTER RACE PCR kit by TakaraBio) as per the manufacturer’s instructions. The consensus sequences for RNA1 (5,851 nucleotide (nt)) and RNA2 (3,468 nt) both exhibited the expected open reading frames (ORFs), and were submitted to NCBI GenBank as RNA1 (PZ055089) and RNA2 (PZ055090). The validated consensus sequences exhibited several base pair variations from the MiSeq sequence throughout the genome (Supplementary data 2). 3’ RACE PCR confirmed the 3’ ends of RNA1 and RNA2 were the same as the MiSeq sequence, however MiSeq did not identify the 5’ ends of either RNA. 5’ RACE PCR added 13 bases to the MiSeq sequence in RNA2. 5’ RACE PCR revealed that the bioinformatic analysis of MiSeq had added an extra 161 nt to RNA1 that were non viral. The validated sequences were aligned to the NCBI database using BLASTn standard databases, megablast ( [1]) and shared ~ 94–96% identity over ~ 95–100% cover with a fabavirus reported from dahlia in China, strain BJ, RNA1 (MN253486.1) and RNA2 (MN253487.1) respectively. The next closest alignments were ~ 70% identity over 47–56% cover with Mikania micrantha mosaic virus (RNA1, EU747708.1, NC_011190.1; RNA2, NC_011189.1; EU719113.1), isolates of gentian mosaic virus.

The BJ sequences (MN253486.1, MN253487.1) were deposited on NCBI in 2017 by Li, Y., where they are described as ‘unpublished’, and represent the first report of this virus in dahlia. The accessions were marked as complete cDNAs and known motifs and domains were highlighted, consistent with a conserved domain (CDD) search having been undertaken. Functional proteins within the polyprotein sequences were not highlighted, which suggested an incomplete molecular characterisation.

Alignment of the BJ sequences (MN253486.1, MN253487.1) with the full-length sequences identified from this study (PZ055089, PZ055090) revealed differences between the RNA1 sequences. The BJ RNA1 (MN253486.1) sequence had 290 fewer nucleotides at the 5’end. The polyprotein start of BJ RNA1 (MN253486.1) was MEKSEL at aa position 59 in comparison to the sequence from this study which started MSSDLA at aa position 1, with numbering based on polyprotein start point from PZ055089. It is unknown if 5’ RACE-PCR was undertaken to confirm the 5’ end of the BJ RNA1 sequence as there was no associated publication, and so it is unclear if this sequence represents an incomplete cDNA or an isolate with a different length and start site.

Analysis of the sequences identified in this study revealed RNAs exhibiting the expected ORFs. A Conserved Domain Search (Wang, 2023 [25]; Lu, 2023 [16]; Marchler-Bauer, 2017 [17]) returned similarities to the following known viral motifs and domain in the RNA1 sequence, RNA_helicase (pfam00910) and Secoviridae_RdRp (cd23196); and in the RNA2 sequence, Como-LCP (pfam02247), and Como_SCP (pfam02248).

Cleavage sites in the proposed polyproteins of the PZ055089 and PZ055090 sequences were predicted by aligning them with accepted and well described full-length *Fabavirus* sequences (Supplementary data 3), while referencing ICTV known fabavirus cleavage site dipeptides (ICTV, [13]). The genome organisation of this dahlia virus (Fig. 1) is consistent with other *Fabavirus* species. The inferred genome is bi-partite, with RNA1 encoding a large polyprotein (1855 aa) that is predicted to be cleaved into a protein cofactor (Co-Pro), helicase (Hel), viral protein genome-linked (VPg), protease (Pro) and RNA-dependant RNA polymerase (Pol). RNA2 codes for a smaller polyprotein (1027 aa) that is predicted to be cleaved into a movement protein (MP), large coat protein (LCP) and a small coat protein (SCP). The same cleavage sites were identified for the BJ sequences.

**Fig. 1:**
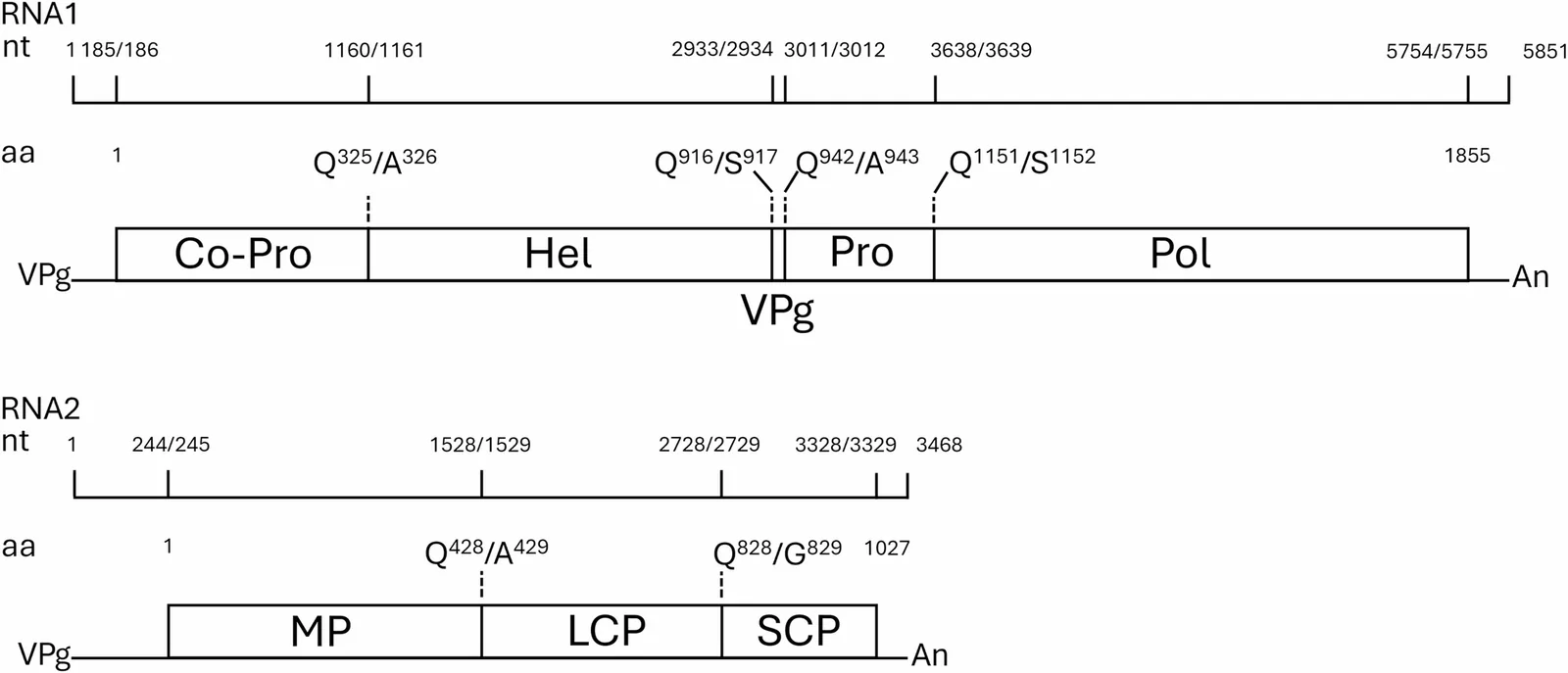
Schematic representation of RNA1 and RNA2 of a fabavirus identified in UK dahlia. The first row of numbers denotes the nucleotide (nt) position, the second row denotes the amino acid (aa) position and also shows the predicted cleavage sites with the aa code. RNA1 and RNA2 both have a VPg at the 5’ end and polyA tails at the 3’ end. RNA1 is 5851 nt long excluding the polyA tail, has a 185 nt 5’UTR and a 97 nt 3’UTR. RNA1 codes for a single large polyprotein that is 1855 aa long and has a predicted molecular weight of 206.7 kDa. Potential cleavage sites have been predicted in RNA1 that create five mature proteins including protein cofactor (Co-Pro), helicase (Hel), viral protein genome-linked (VPg), protease (Pro) and RNA-dependant RNA polymerase (Pol). The predicted cleavage sites are Co-Pro/Hel Q325/A326, Hel/VPg Q916/S917, VPg/Pro Q942/A943 and Pro/Pol Q1151/S1152. The predicted sizes and predicted molecular weights of the mature proteins are Co-Pro 325 aa and 35.75 kDa; Hel 591 aa and 65.54 kDa; VPg 26 aa and 3.09 kDa; Pro 209 aa and 23.15 kDa and Pol 704 aa and 79.24 kDa. RNA2 is 3468 nt long excluding the polyA tail, has a 244 nt 5’UTR and a 140 nt 3’UTR. RNA2 codes for a single smaller polyprotein that is 1027 aa long and has a predicted molecular weight of 115.21 kDa. Potential cleavage sites have been predicted in RNA2 that create three mature proteins including protein movement protein (MP), large coat protein (LCP) and the small coat protein (SCP). The predicted cleavage sites are MP/LCP Q428/A429, LCP/SCP Q828/G829. The predicted sizes and predicted molecular weights of the mature proteins are MP 428 aa and 48.07 kDa; LCP 400 aa and 44.16 kDa; SCP 199 aa and 23.02 kDa. The total length of RNA1 is 3468 nucleotides not including the polyA tail

To determine the relationships of the UK (PZ055089, PZ055090) and the BJ (MN253486.1, MN253487.1) isolates to related viruses we compared them to 145 members of the *Secoviridae* using the ProPol sequence region (Supplementary data 4). Sequences were aligned using ClustalW and the phylogenetic tree was generated using MEGA 12 with the Maximum Likelihood method [7, 14, 15, 23]. This tree showed that both viruses sat with other fabavirus sequences. Alignments were undertaken and phylogenetic trees generated for RNAs 1 (Fig. 2a) and 2 (Fig. 2b) of a range of well described fabaviruses. Data shown in Fig. 2 indicates that the dahlia fabavirus sequences were representative of a novel species and not a strain of an existing accepted fabavirus.

**Fig. 2:**
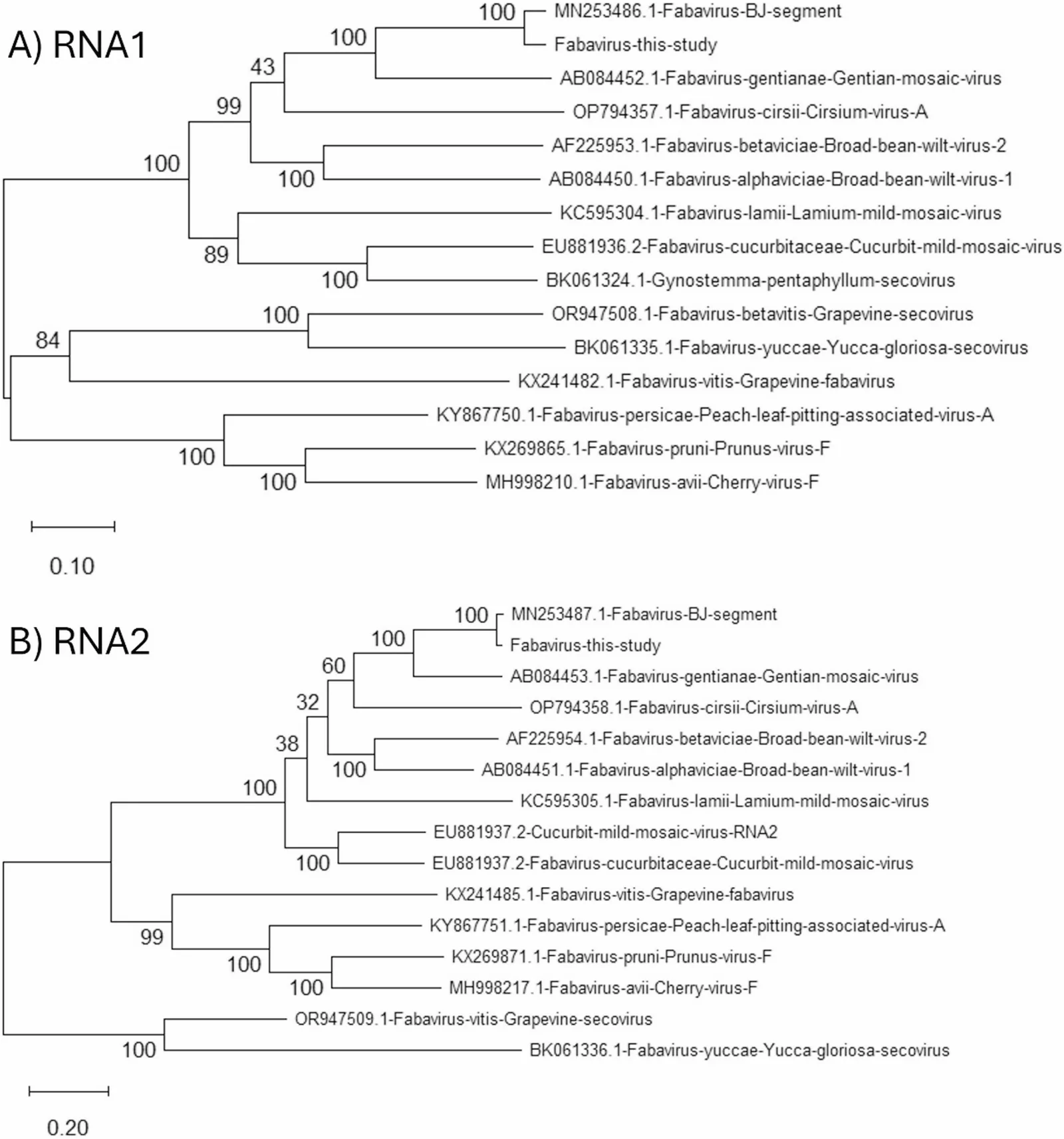
Phylogenetic trees comparing ***Fabavirus*** RNA1 nucleotide sequences (**A**) and ***Fabavirus*** RNA2 nucleotide sequences (**B**). The virus sequences compared in these analyses were chosen based on them being *Fabavirus* member species noted by ICTV. The sequences were aligned using ClustalW and the trees were generated in Mega 12 (Kumar, 2024 [15]), using the Maximum Likelihood method [7, 23, 24] with 1000 bootstrap replicates. UK (Fabavirus, this study PZ055089, PZ055090) and BJ (MN253486.1, MN253487.1) sequences shared a branch in both RNA1 and RNA2 comparisons, with gentian mosaic virus sitting on the adjacent branch

The ICTV species demarcation thresholds for *Secoviridae* are 80% aa identity over the Pro-Pol region and 75% aa identity over the CP regions (individually or combined). The percentage identities in comparison to the closest described fabavirus gentian mosaic virus (RNA1 NC_077675.1, RNA2 NC_077674.1) were 78.2% over the Pro-Pol region (under the 80% aa identity threshold); 72.9% over the LCP and 74.3% over the combined coat protein regions (under the 75% aa identity threshold). The SCP crossed the threshold by 0.4% compared to gentian mosaic virus (RNA2 NC_077674.1). These comparisons suggested that the sequence data presented here along with the BJ sequence represent a novel species of *Fabavirus* that infects dahlia.

No recombination events were identified by two or more methods using the full suite of options on default in RDP4 [18] when the dahlia fabavirus (PZ055089, PZ055090; MN253486.1, MN253487.1) and gentian mosaic virus (NC_077675.1, NC_077674.1) sequences were compared.

Widespread use of HTS platforms have led to large amounts of sequence data being submitted to GenBank/NCBI for newly described viruses [8, 9]. This study highlights the requirement to validate such proposed viral sequences using RT-PCR and RACE PCR and to analyse and annotate them. The HTS-inferred RNA1 sequence was longer than the validated sequence (PZ055089) by 161 nt. These extra nt (not present in 5’ RACE products) were identified as being of chloroplast origin when compared to the NCBI database. Validation with 5’RACE PCR also added 13 bases to the 5’ end of the HTS-inferred RNA2 sequence. The coding start point was the same for HTS-inferred and validated sequences.

The ICTV Pro-Pol, LCP and combined CP species demarcation criteria relating to sequence data were satisfied for the dahlia fabavirus sequences (PZ055089, PZ055090; MN253486.1, MN253487.1) to be considered a new species. The name dahlia fabavirus (DFV) is proposed for this species with suggested species name: “*Fabavirus dahliae*”.

## Supplementary Information

Below is the link to the electronic supplementary material.

Supplementary Material 1 (DOCX 16.9 KB)

Supplementary Material 2 (DOCX 18.6 KB)

Supplementary Material 3 (DOCX 15.6 KB)

Supplementary Material 4 (PDF 122 KB)

## Data Availability

Sequence data generated has been submitted to NCBI Genbank with the following accession numbers PZ055089 and PZ055090. Primer sequences and sequence comparisons are provided in supplementary data, and raw sequence data is available in SRA and linked to the Genbank accessions.
