# Supplementary material for: Sequence analysis of a viral strain isolated for the first time in the UK, clarifies the identity of a novel species of fabavirus
Source: Arch Virol. 2026 Jul 15;171(8):226. doi: 10.1007/s00705-026-06690-6 (PMC13372865; doi:10.1007/s00705-026-06690-6)
Supplement: Supplementary file 1 — Supplementary Material 1 (DOCX 16.9 KB) [file 705_2026_6690_MOESM1_ESM.docx]

**Supplementary data 1**

Table 1.1. Primers used in sequence validation

| **Primer ID** | **Sequence** | **Region** |
| --- | --- | --- |
| F1-5R-v4 | GCATGCGCCTTCAAAATTGCAAATCGAG | RNA1, 5' RACE |
| 5 | CGAGACAATCCCAACACCCA | RNA1 |
| 6 | TGGCTCCATTATGCGTTGGT | RNA1 |
| 15 | GAGCAGGCATTCGCGTGG | RNA1 |
| 16 | GACTTCATTAGGACCCAGCTG | RNA1 |
| 17 | CAAGGAAGGCGCCATTTATGG | RNA1 |
| 19 | GTCACGTGTGATCTAGGTGTTG | RNA1 |
| 20 | GCATCTGGACACGCCTTGC | RNA1 |
| 21 | CTCGCACCATTGGGACATCC | RNA1 |
| 26 | GCATGCGCCTTCAAAATTGC | RNA1 |
| 33 | GACGCCAATAGCTTGGCTATAG | RNA1 |
| 35 | CAAGCTGGTCCTGCTATTGTG | RNA1 |
| 36 | GATTCTAGGCAGACCATCAGC | RNA1 |
| 40 | GAGACCATACCTGGCACAGCTC | RNA1 |
| 44 | TTCTACGTCCTTTCTGCGGG | RNA1 |
| 1A | GTCATGCCCACTGCAAAAGG | RNA1 |
| 22F | GTTCCCTTTGACGGTGATAGTG | RNA1 |
| 23R | CTACCACGATCCCTGGCAG | RNA1 |
| 24F | CGATCAAATGACTGGCACAG | RNA1 |
| 2A | GTGTGCAGGTTTGCATGATGC | RNA1 |
| 34NEW | CCTTTAGGGCCACAACACC | RNA1 |
| FAB-R1-3' | GTGGGCGTGGTGGTCTCCCCACGAAG | RNA1, 3' RACE |
| FAB-RNA1-3' | ATATAATCACACAAATACTTTAAACATAAAGATC | RNA1 |
| FAB-R2-5' | GAACTGGCGTTCCATCTCTTCCGCTGTG | RNA2, 5' RACE |
| FAB-RNA2-5' | ATTTAAACAAACAGCTTTCGTCGGATAAAACAG | RNA2 |
| 7 | CAGCTTTCGGTTCTGGACTTC | RNA2 |
| 8 | GTCAGGACCCTTGGCCTTTG | RNA2 |
| 9 | GAGTTCCATCCACAGATGATCC | RNA2 |
| 27 | TCAACGCTTGGCCACACTTA | RNA2 |
| 28 | AGCTTCACACTGGCAACACT | RNA2 |
| 9 | GAGTTCCATCCACAGATGATCC | RNA2 |
| 30 | CAAGATTTGGATGAAGGTCTAGG | RNA2 |
| 10 | GCACACAAATTCTATAGCAGGCTC | RNA2 |
| 11 | GTCCTGCAAATGGGATAGGAC | RNA2 |
| 31 | CATGAGGCAAACAATCCAAGG | RNA2 |
| 32 | CCAAGGGGTTAGGAAAGCGT | RNA2 |
| FAB-CP-2 | GTGTAGCAACCGGGTGAAG | RNA2 |
| 4A | GCGCTGCGCAGGTGAGATTG | RNA2 |
| 3A | GAGGAAAGCACACCCCACAC | RNA2 |
| 13 | CACACTGGATCAGGAGG | RNA2 |
| FAB-R2-3' | GTCAATGGGCTGGGATGTGCAAGGAG | RNA2, 3' RACE |
| FAB-RNA2-3' | TTTTTATATAAACACACCAACGAAATC | RNA2 |
