# Supplementary material for: Sequence analysis of a viral strain isolated for the first time in the UK, clarifies the identity of a novel species of fabavirus
Source: Arch Virol. 2026 Jul 15;171(8):226. doi: 10.1007/s00705-026-06690-6 (PMC13372865; doi:10.1007/s00705-026-06690-6)
Supplement: Supplementary file 2 — Supplementary Material 2 (DOCX 18.6 KB) [file 705_2026_6690_MOESM2_ESM.docx]

**Supplementary data 2**

Supplementary data Table 2.1 Base pair variations between MiSeq and consensus sequences

| FAB1 | 4914 | 4929 | 4950 | 4959 | 4974 | 5019 | 5026 | 5040 | 5052 | 5059 | 5089 |
| --- | --- | --- | --- | --- | --- | --- | --- | --- | --- | --- | --- |
| MiSeq | G | T | A | C | C | A | T | C | C | T | C |
| Clones | A 2 | 2 C | 2 T | 2 T | 2 T | 2 G | 2 C | 2 T | 2 T | 2 C | 2 T |
| Consensus | A | C | T | T | T | G | C | T | T | C | T |
| Coding change? | N | N | E/D | N | N | N | W/R | N | N | N | N |
|  |  |  |  |  |  |  |  |  |  |  |  |
| FAB1 | 5097 | 5100 | 5109 | 5121 | 5148 | 5193 | 5200 | 5424 | 5430 | 5431 |  |
| MiSeq | T | C | G | T | G | G | C | A | A | T |  |
| Clones | 2 C | 2 T | A 2 | 2 C | A 2 | A 2 | 2 T | 2 G | 2 G | A 2 |  |
| Consensus | C | T | A | C | A | A | T | G | G | A |  |
| Coding change? | N | N | N | N | N | N | N | N | N | S/T |  |
|  |  |  |  |  |  |  |  |  |  |  |  |
| FAB2 | 201 | 213 | 214 | 279 | 284 | 287 | 371 | 455 | 460 | 476 | 487 |
| MiSeq | A | G | C | A | C | A | T | T | T | A | G |
| Clones | 4 G | : 4 | 4 T | 4 T | 4 T | 4 G | 4 C | 3 C | 3 C | 3 T | A 3 |
| Consensus | G | : | T | T | T | G | C | C | C | T | A 3 |
| Coding change? | N (utr) | N/A | N | I/F | N | N | N | N | V/A | E/D | R/K |
|  |  |  |  |  |  |  |  |  |  |  |  |
| FAB2 | 1310 | 1319 | 1337 | 2345 | 2358 | 2369 | 2600 | 2603 | 2604 | 3157 | 3167 |
| MiSeq | C | C | A | C | G | A | T | : | : | : | : |
| Clones | 2 T | 2 T | 2 G | 2 T | A 2 | 2 T | T/G | :/T | :/T | :/T | :/T |
| Consensus | T | T | G | T | A | T | T | : | : | : | : |
| Coding change? | N | N | N | N | V/I | N | N | N | N | N | N |
|  |  |  |  |  |  |  |  |  |  |  |  |
| FAB2 | 3276 | 3284 | 3286 | 3289 | 3298 | 3360 | 3473 | 3475 |  |  |  |
| MiSeq | A | T | A | A | A | T | T | T |  |  |  |
| Clones | A/G | T/G | A/: | A/: | A/G | T/G | A/T | A/T |  |  |  |
| Consensus | A | T | A | A | A | T | T | T |  |  |  |
| Coding change? | K/E | N | N | N | D/G | UTR | UTR | UTR |  |  |  |
