# Supplementary material for: Sequence analysis of a viral strain isolated for the first time in the UK, clarifies the identity of a novel species of fabavirus
Source: Arch Virol. 2026 Jul 15;171(8):226. doi: 10.1007/s00705-026-06690-6 (PMC13372865; doi:10.1007/s00705-026-06690-6)
Supplement: Supplementary file 3 — Supplementary Material 3 (DOCX 15.6 KB) [file 705_2026_6690_MOESM3_ESM.docx]

**Supplementary data 3**

Supplementary data Table 3.1 Accepted fabavirus sequences referred to in analysis.

| **Virus** | **RNA 1 NT** | **RNA 1 AA** | **RNA 2 NT** | **RNA 2 AA** |
| --- | --- | --- | --- | --- |
| *Fabavirus alphaviciae* (broad bean wilt virus 1) | AB084450 | BAD00183.1 | AB084451 | BAD00184.1 |
| *Fabavirus avii* (cherry virus F) | MH998210 | AZZ10048.1 | MH998217 | AZZ10055.1 |
| *Fabavirus betaviciae* (broad bean wilt virus 2) | AF225953 | AAK27841.1 | AF225954 | AAK27842.1 |
| *Fabavirus betavitis* (grapevine secovirus) | OR947508 | WRQ19773.1 | OR947509 | WRQ19774.1 |
| *Fabavirus cirsii* (cirsium virus A) | OP794357 | WCR31000.1 | OP794358 | WCR31001.1 |
| *Fabavirus cucurbitaceae* (cucurbit mild mosaic virus) | EU881936 | ACK76423.1 | EU881937 | ACK76424.1 |
| *Fabavirus gentianae* (gentian mosaic virus) | AB084452 | BAD99001.1 | AB084453 | BAD99002.1 |
| *Fabavirus gynostemmae* (gynostemma pentaphyllum secovirus) | BK061324 | DAZ91079.1 | BK061325 | DAZ91066.1 |
| *Fabavirus lamii* (lamium mild mosaic virus) | KC595304 | AHB38892.1 | KC595305 | AHB38893.1 |
| *Fabavirus persicae* (peach leaf pitting-associated virus A) | KY867750 | ATD53314.1 | KY867751 | ATD53315.1 |
| *Fabavirus pruni* (prunus virus F) | KX269865 | ANH71247.1 | KX269871 | ANH71253.1 |
| *Fabavirus vitis* (grapevine fabavirus) | KX241482 | ANG55944.1 | KX241485 | ANG55947.1 |
| *Fabavirus yuccae* (yucca gloriosa secovirus) | BK061335 | DAZ91075.1 | BK061336 | DAZ91076.1 |
