# Supplementary figures and images for: Sequence analysis of a viral strain isolated for the first time in the UK, clarifies the identity of a novel species of fabavirus
Source: Arch Virol. 2026 Jul 15;171(8):226. doi: 10.1007/s00705-026-06690-6 (PMC13372865; doi:10.1007/s00705-026-06690-6)

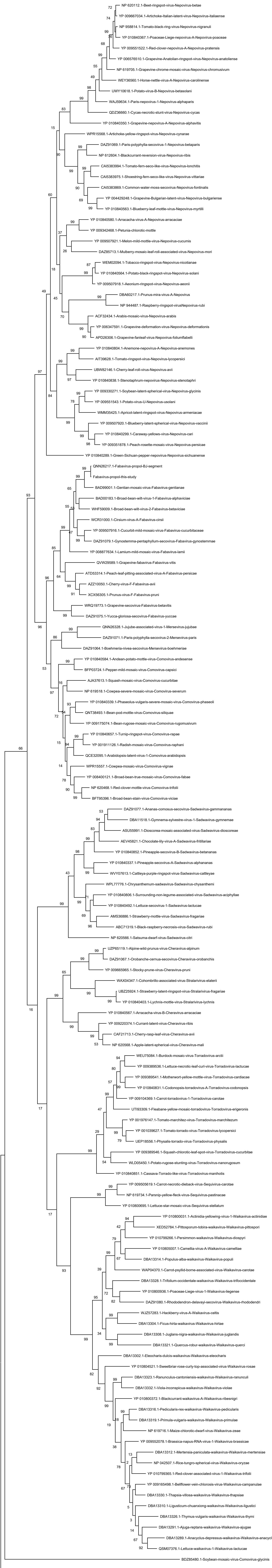

0.50

Supplement: Supplementary file 4 — Supplementary Material 4 (PDF 122 KB) [file 705_2026_6690_MOESM4_ESM.pdf]
